# Data-Driven Analysis of Bicycle Sharing Systems as Public Transport Systems Based on a Trip Index Classification

**DOI:** 10.3390/s20154315

**Published:** 2020-08-02

**Authors:** Mark Richard Wilby, Juan José Vinagre Díaz, Rubén Fernández Pozo, Ana Belén Rodríguez González, José Manuel Vassallo, Carmen Sánchez Ávila

**Affiliations:** 1 Group Biometry, Biosignals, Security, and Smart Mobility, Departamento de Matemática Aplicada a las Tecnologías de la Información y las Comunicaciones, Escuela Técnica Superior de Ingenieros de Telecomunicación, Universidad Politécnica de Madrid, Avenida Complutense 30, 28040 Madrid, Spain; mrwilby@etsit.upm.es (M.R.W.); ruben.fernandez@upm.es (R.F.P.); abrodriguez@etsit.upm.es (A.B.R.G.); carmen.sanchez.avila@upm.es (C.S.Á.); 2 Grupo de Investigación en Planificación del Transporte, Transport Research Centre (TRANSyT), Escuela Técnica Superior de Ingenieros de Caminos, Canales y Puertos, Universidad Politécnica de Madrid, 28040 Madrid, Spain; josemanuel.vassallo@upm.es

**Keywords:** bicycle sharing systems, public transport systems, data-driven classification of trips, BSS underlying network, trip index

## Abstract

Bicycle Sharing Systems (BSSs) are exponentially increasing in the urban mobility sector. They are traditionally conceived as a last-mile complement to the public transport system. In this paper, we demonstrate that BSSs can be seen as a public transport system in their own right. To do so, we build a mathematical framework for the classification of BSS trips. Using trajectory information, we create the *trip index*, which characterizes the intrinsic purpose of the use of BSS as *transport* or *leisure*. The construction of the trip index required a specific analysis of the BSS shortest path, which cannot be directly calculated from the topology of the network given that cyclists can find shortcuts through traffic lights, pedestrian crossings, etc. to reduce the overall traveled distance. Adding a layer of complication to the problem, these shortcuts have a non-trivial existence in terms of being intermittent, or short lived. We applied the proposed methodology to empirical data from BiciMAD, the public BSS in Madrid (Spain). The obtained results show that the trip index correctly determines transport and leisure categories, which exhibit distinct statistical and operational features. Finally, we inferred the underlying BSS public transport network and show the fundamental trajectories traveled by users. Based on this analysis, we conclude that 90.60% of BiciMAD’s use fall in the category of transport, which demonstrates our first statement.

## 1. Introduction

Bicycle sharing systems (BSSs) are one illustrative example of the new sharing economy, which is exponentially increasing at present, with great impact on mobility [1]. At first instance, they have arisen as a perfect complement for public transport in cities, covering the last-mile segment [2]. In this respect, BSSs provide the convenience of unidirectional short hops, which enhance the connections to other transport modes: bus, metro, train, etc. Under this perspective, BSSs are just conceived as supplementary to the main transport network. However, BSSs can also be considered a transport mode in their own right, which contributes to the overall objective of reducing car use [3]. This reduction implies a direct positive impact on environment and health [4].

BSSs show specific characteristics that make them different from most transport modes: their use. Cars, buses or trains are primarily used as a means of moving people from one place to another. These trips are performed to reach the workplace, the residence, a commercial area, a sport center, or a tourist site, among others. In this respect, we can observe that the purpose of these trips is mainly defined by their destination. BSSs are fundamentally different as the users’ purpose is inherent to the trip: not only can they be performed to reach a destination, but also trips themselves can be a form of sport or tourism, for example [5].

With greater understanding of their usage patterns, BSSs could be optimized, tailoring them to each city and neighborhood. The current knowledge about BSS usage is still embryonic and the need to identify sub-modes of the different mobility activities taken by BSS users is an essential step in this process. However, as our understanding increases, so will our design criteria and planning. BSSs can be re-purposed and redeployed with very little capital investment and virtually no infrastructure change. These small budget requirements will allow the expansion of BSSs, which offers a high degree of granularity, making them an extremely useful tool in the upgrading of mobility services.

Human mobility is a complex problem. Fortunately, some aspects of this complexity assist in the problem analysis: (i) people move according to regular patterns [6]; and (ii) they return to a small number of places [7]. To move, people choose public transport or private vehicles considering measurable factors, such as travel and transit times or economic cost [8], and other non-measurable factors, like convenience, safety, comfort, or environment [9].

This extensive set of factors complicates the task of modeling human mobility and, in particular, the election of a BSS as a transport mode. In the absence of data, we must rely on probabilistic methods [10]. However, in this case, although not complete, we do have access to some information. BSSs often provide data regarding the occupancy of docking stations, which we can employ to infer the demand between them [11]. From this information we can then extract a set of behavioral profiles for each docking station, such as *morning peak*, *morning arrival*, *flat* or *anomalous* [12].

Presently, 4th generation BSSs provide trajectory data, which allow us to construct more accurate profiles [13]. These trajectories can also be extracted from other by-products like call detail records (CDR) collected by phone companies [14]. However, this approach is often biased by the specific population that formed the dataset [15].

In this work we analyze trajectory data from BiciMAD, a 4th generation BSS in Madrid, Spain, and create a data-driven methodology to classify trips into two main groups depending on their intrinsic purpose: transport and leisure. We understand as *transport trips* those performed to reach a specific destination in the shortest possible time. This reflects that the main purpose of this category of trips is getting to the destination to develop certain activity like working, studying, shopping, etc. On the other hand, we understand as *leisure trips* those performed to wander around the city or as sport. In this respect, the fundamental objective of leisure trips is not the destination itself, but the route traveled along a sequence of points of interest (tourism) or the overall length of the path (sport).

In addition, this methodology allows us to infer the underlying public transport network of a BSS. Most transport modes are composed by a permanent set of nodes (stations or stops) and a structure of fixed edges [16], i.e., the specific routes of buses or trains. On the contrary, a BSS has no fixed edges given that users can cycle following whichever route they prefer through the road system, parks, bicycle lanes, etc. This makes the extraction of the underlying transport network a challenging task. Our methodology provides a rigorous way of inferring the BSS transport network from trajectory data, thus contributing to reach a complete understanding of the overall multiplex public transport network [17] of a city.

Consequently, the main contributions of this work are:

- The creation a quantitative framework to classify BSS trips as *transport* or *leisure*.
- The definition of a distance-based index that builds the basis for this classification of trips.
- The mathematical characterization of the shortest path distance in a BSS, considering the set of shortcuts that bikers can use in their routes.
- The application of this framework to classify trips in a real BSS.
- Statistical and operational analysis to confirm the validity of the obtained results.
- The extraction the underlying BSS public transportation network.

In the following sections we first propose a mathematical framework to classify BSS trips (Section 2). Next, in Section 3 we present and validate the results of applying this framework to a real dataset, providing the methodology we used to calculate the different variables it involves. From the basis of the resulting classification of trips, Section 4 shows the underlying transport and leisure networks we extract from the real trajectories falling on each category. Finally, Section 5 summarizes the work and discusses its benefits for municipalities and BSS managers. Please note that this structure does not include a specific section devoted to analyzing scientific works in the field. Instead we have placed each reference in the particular section that required it. This way the reader can easily follow the text eliminating the need for returning to a previous section to check each reference.

## 2. Data-driven Classification of Trips

The present section is devoted to describing the mathematical framework that builds the basis of the method of trip classification we propose. First (Section 2.1) we set up the starting premise upon which we will construct the trip classification methodology. This starting premise is based on observing trajectories and detecting how close they are to the shortest path. Then we create a trip index as the fundamental metric, which compares the actual trajectory to a reference (Section 2.2). However, this reference is not unique as BSS users can find shortcuts to reduce their overall traveled distance; thus, we study the resulting spaces and trajectories in Section 2.3. Finally, in Section 2.4 we define the shortest path applicable to a BSS and the subsequent trip index.

### 2.1. Starting Premise

Our first purpose is to construct a methodology and a mathematical framework that allow us to classify BSS trips depending on their intrinsic purpose. In this respect, we can observe four types of BSS users [5]: (a) commuters: who move between residence and work place, or secondary transport node; (b) utility users: who need to reach commercial, cultural or sport facilities; (c) leisure users: who cycle for fun and sport; and (d) tourists: who visit tourist sites and attractions. To reach our main objective, we define two categories of BSS trips: *transport*, which includes group types (a) and (b); and *leisure*, which include group types (c) and (d). Please note that we do not classify *users*, but *trips* as our final goal is to characterize the BSS mobility and its intrinsic network. In this sense, a determined user can always perform trips that fall in either category.

A trip is a sequence of locations and time stamps. From this set of spatio-temporal points we can obtain different variables that describe the trip, like speed and length. Considering the former, we could expect the speed of leisure trips to be lower than that of transport trips. However, speed may not be adequate for this purpose as it depends on factors like age, physical abilities, steepness of the road, or traffic. On the other side, the length of the trip is intrinsically impacted by the separation between origin and destination, thus it cannot directly reflect the purpose of the journey as an absolute value.

So, let us observe the trajectory of the trip. Research in public transportation systems assume that users apply some utility function to their decision to choose this transport mode [18,19]. Consequently, we build our framework based on this starting premise: transport trips describe trajectories close to the shortest path.

### 2.2. Trip Index

Our objective is to define a *trip index* α that allows us to classify trips into transport or leisure. To calculate this index, we will rely on trajectories. In this respect, note that trajectories do not need to be identical to describe a specific type of trip; they just have to share some common semantic meaning, which in our case, will be given by the trip index. Following our starting premise, the trip index must represent the deviation of the user’s actual trajectory from the shortest path.

In other scientific disciplines, for example Biology and Hydraulic, researchers have faced similar problems in describing animal’s movements searching for food [20] and the course of rivers through valleys [21] respectively. These works make use of the *sinuosity index*, SI, which relates the distance of the linear and actual paths:

$$SI=\frac{d_{p}}{d_{L}},$$

where $d_{p}$ is the length of the actual trajectory and $d_{L}$ is the Euclidean distance between origin and destination.

However, linear trajectories are not viable in cities most of the time, because of the intrinsic topology of the infrastructure. Thus, we must take the shortest possible path as the base reference. Considering these premises, we define the trip index $\alpha_{p}$ of a trip *p* from origin *i* to destination *j* as:

(1) $$\alpha_{p}=\frac{d_{SP}\left(i,j\right)}{d_{p}},$$

where $d_{p}$ is the actual distance traveled in trip *p* and $d_{SP}$ is the length of the shortest viable path from origin to destination. However, the peculiarities of a BSS insert a level of complexity in calculating this shortest path.

### 2.3. Spaces, Trajectories and Shortcuts in a BSS

To represent trips in a BSS system, we must consider two spaces. On one hand, BSSs are deployed on a *real physical space*, with constraints. Some of these constraints are immutable, such as buildings or rivers; and others are subject to change, like one-way streets or shared lanes (which cars and bicycles can simultaneously use). On the other hand, this real physical space with constraints defines an *underlying graph*, which represents the actual allowed travel space. The underlying graph continuously evolves in time as it is affected by mutable constraints.

In addition, given the particularities of BSSs, we have to contend with the fact that the underlying graph is not complete, and we must also consider missing connections in the real space. This is because cyclists can navigate through some restrictions using, for example, pedestrian crossings or sidewalks. We will collectively refer to these extra graph elements as *shortcuts*. Furthermore, the existence of these shortcuts has a complex time dependency as they may be impacted by factors that make them viable or not at specific moments. For example, consider the possibility of crossing a street on a traffic light; only when it is green, this shortcut becomes a viable alternative to reduce the minimum distance. Thus, shortcuts can be observed as graph edges with a time-to-live.

Considering these premises, we can define four different trajectories regarding a determined BSS trip:

- The linear trajectory, with length $d_{L}$, i.e., the direct Euclidean distance between origin and destination, which only depends on the real physical space.
- The retrievable shortest path between origin and destination given the underlying graph, which we will refer to as the *orthodox* trajectory, with length $d_{O}$.
- The shortest path between origin and destination using the set of available shortcuts, namely the *heterodox* trajectory, with length $d_{H}$.
- The actual path of the trip traveled by the user, with length $d_{p}$.

### 2.4. Characterizing the Shortest Path in a BSS Trip

Given a trip *p*, the Euclidean distance $d_{L}$ from origin *i* to destination *j* defines the length scale to the problem: the relative size of graph links to the Euclidean distance from origin to destination.

On the other hand, the orthodox trajectory is the one that minimizes the overall distance traveled from origin to destination, using the corresponding city infrastructure (streets and bicycle lanes) and respecting the regulation applicable to bicycles. This orthodox trajectory does not embed the heterodoxy that results from inserting shortcuts in the graph, which effectively reduces $d_{O}$.

In addition, we must take into account that these shortcuts evolve in time. This variation implies that we cannot aim at defining a *fixed* shortest path, but a *set of viable* shortest paths. Effectively, we need some way of characterizing the statistical distribution of this set of available shortest paths.

The problem of directed paths is intrinsic to a large number of different disciplines, polymer physics in the presence of forces, percolation theory, direct random walks, to name but a few. Using some of the more basic concepts from these fields we can infer what the basic structure of the probability distribution of shortest paths could be. This distribution function is built from a collection of paths fixed to have the same start and end points, which are separated by $d_{L}$. In effect, we are considering a distribution function constructed from a set of paths defined by a fixed extension. The actual distribution will be complex, where details of its form will depend on the nature of the paths available, but we are proposing that the grosser characteristics are defined by only a limited number of parameters. The simplest spatial representation of this statistical distribution of paths is that of an ellipsoid with origin and destination located on its vertexes. The width of the ellipsoid is dictated by allowed variance and frequency of deviations from the direct path. Consequently, we obtain a crude characterization of this distribution by looking at the defining geometry of the ellipse. Hence, the solution to our problem revolves around the calculation of the major and minor axes of this ellipsoid. The former is the linear trajectory, with length $d_{L}$. Therefore, let us concentrate on the latter.

The minor axis reflects the distribution width caused by deviations from the linear path of the actual trip. We can find a set of approaches within the scientific literature in the field of urban mobility that estimate deviations from trajectories using map-matching methods or path-searching problems [22]. These approaches often aim at coupling a set of GPS data points to one of the possible trajectories a vehicle can describe through the underlying city’s graph. Among them, researchers have used methods like the $A^{☆}$ search algorithm [23], the Manhattan Distance [24], or the Hausdorff distance [25].

However, our problem is intrinsically different as we need to characterize the distribution function of a set of viable and time-dependent shortest paths. This set of shortest paths presents an absolute minimum, the linear path. Consequently, we can evaluate deviations using a similarity metric to the linear path [26]. For this purpose, we chose the Fréchet distance [27], which measures the similarity between two curves, considering the location and order of points along them. Intuitively, imagine a person walking a dog with a leash. The person and the dog describe finite paths *f* and *g* respectively. Both can vary their speed, but they cannot walk back. The Fréchet distance between curves *f* and *g* could be seen as the minimum length of the leash that allows these two trajectories.

Formally [28], let (M,d) be a metric space; we define a *curve* as a continuous mapping:

$$f:\left[v_{0},v_{1}\right]\to M,\;v_{0},v_{1}\in I\;R,\;\;v_{0}\leq v_{1}.$$

Given two curves $f:[v_{0},v_{1}]\to M$ and $g:[w_{0},w_{1}]\to M$, their *Fréchet distance* is defined as:

δF(f,g)=infv:[0,1]→[v0,v1]w:[0,1]→[w0,w1]maxt∈[0,1][dfv(t),gw(t),

where *v* and *w* are arbitrary continuous nondecreasing functions, with $v\left(0\right)=v_{0}$, $v\left(1\right)=v_{1}$, $w\left(0\right)=w_{0}$, and $w\left(1\right)=w_{1}$ and *t* is the path parameter, typically time.

For trajectories formed as a collection of points rather than a continuous function, we can use the discrete Fréchet distance, also called the *coupling distance* [29]. It is an approximation of the Fréchet metric for polygonal curves. Following a similar intuitive example mentioned before, the discrete Fréchet distance replaces the man and the dog with a pair of leaping frogs.

Using the formal definition in [29], let *P* and *Q* be two polynomial curves with endpoints of their line segments forming the sequences $\sigma \left(P\right)=\left(v_{1},v_{2},\ldots ,v_{p}\right)$ and $\sigma \left(Q\right)=\left(w_{1},w_{2},\ldots ,w_{q}\right)$. A coupling *C* between *P* and *Q* is a sequence of distinct pairs of endpoints in σ(P)×σ(Q) taken in order:

$$C=\left(\left(v_{a_{1}},w_{b_{1}}\right),\left(v_{a_{2}},w_{b_{2}}\right),\ldots ,\left(v_{a_{m}},w_{b_{m}}\right)\right).$$

The first and last pairs are formed by the two origins and two destinations, respectively. The remaining pairs are formed in an iterative sequence. In each step, we couple the previous or the next endpoints in σ(P) to the previous or the next in σ(Q). This method implies that there is not a unique coupling; every particular combination of endpoints will generate a coupling $C_{k}$ with k∈[1,K], whose length is determined by the longest distance between every pair of endpoints, i.e.

$$\left|\right|C_{k}\left|\right|=\max_{i=1,2,\ldots ,m}d\left(v_{a_{i}},w_{b_{i}}\right).$$

Finally, the *discrete Fréchet distance* between the polygonal curves *P* and *Q* is defined as the minimum length among all the possible *K* couplings between them:

(2) $$\delta_{DF}\left(P,Q\right)=\min_{k\in [1,K]}\left\{|\right|C_{k}||\}.$$

We use the discrete Fréchet distance to calculate the maximum deviation of the orthodox trajectory to the linear trajectory, i.e., $\delta_{DF}\left(O,L\right)$. Considering this maximum deviation, the length of the heterodox trajectory lies between those two distances, i.e., $d_{H}\in \left[d_{L},d_{O}\right]$. Thus, we define the length of the heterodox trajectory as the minimum distance between origin and destination, given a deviation of $\delta_{DF}\left(O,L\right)$ from the linear trajectory, which is calculated as:

(3) $$d_{H}=2\sqrt{{\left(\delta_{DF}\left(O,L\right)\right)}^{2}+\frac{d_{L}^{2}}{4}}=\sqrt{4{\left(\delta_{DF}\left(O,L\right)\right)}^{2}+d_{L}^{2}}$$

We will take $d_{H}$ as an approach to the shortest available path between the origin *i* and destination *j* of a particular trip *p*. Consequently, the trip index defined in Equation 1 is finally defined as:

(4) $$\alpha_{p}=\frac{d_{H}}{d_{p}}=\frac{\sqrt{4{\left(\delta_{DF}\left(O,L\right)\right)}^{2}+d_{L}^{2}}}{d_{p}}$$

The trip index in Equation 4 incorporates all the available information about a trip and the possible trajectories in the underlying physical and topological spaces of the BSS. Following the premise stated in Section 2.1, we will use the trip index to classify trips as leisure or transport.

## 3. Application to a Real BSS

We applied the trip index to a dataset of real trips performed in BiciMAD, the public BSS in the city of Madrid, Spain. In this section, we will describe the dataset and the methodology we followed to obtain indexes and classify the trips accordingly. Finally, we will analyze the characteristics of the resulting groups of transport and leisure trips to validate the classifier under both statistical and operational perspectives.

### 3.1. Dataset

BiciMAD is a public BSS managed by the Empresa Municipal de Transportes (EMT) within the Municipality of Madrid (Spain). BiciMAD uses electric bicycles and includes 169 docking stations currently in operation.

We used data from February 2019 accounting for near 500MB carrying information corresponding to 303962 trips. Each trip includes the following data:

- Time stamp: pick up time with 1 hour definition, for privacy and anonymity issues.
- User’s identifier: unique encrypted identifier of user, refreshed daily.
- Type of user: annual, eventual, staff.
- User’s range of age: 6 intervals [0,16], [17,18], [19–26], [27–40], [41–65], [66,*∞*), and *unknown*.
- Identifier of the origin docking station.
- Identifier of the destination docking station.
- Travel time: time from pick up to drop off.
- Track: collection of geographical coordinates ordered in time recorded on a 1-minute basis during the trip.

### 3.2. Applying the Mathematical Framework to the Dataset

To apply the mathematical framework, we developed in Section 2 to this dataset, we need to perform a sequence of tasks that we next describe.

#### 3.2.1. Preprocessing

We first carry out a preprocessing phase to guarantee the quality of the data we employ. Errors in the database are mainly due to the low accuracy of GPSs in an urban scenario, which results in either inconsistent positions or time slots with no recorded location. Furthermore, there are trips performed by staff members that we do not consider in the study.

We apply this preprocessing to 303962 trips connecting 25818 origin and destination pairs resulting in 142968 movements and 22929 origin-destination (OD) pairs.

In addition, we filter out those trips that start and end on the same docking station as they cannot be considered to be transport. In this filtering we treat as *twin* docking stations those that are located at the same point of interest. These trips follow their own internal dynamics and are also intention-based. They are possibly retrieval trips (target destination and return). They could also be tourist/leisure-based activity, or they could just represent an interpretation of the tariffing, where the user considers it more effective to retain the bike for whatever reason. Such paths should be considered separately, but with no knowledge of the destination, they cannot be used to analyze the transport aspects of the service. In any case, this restriction forces us to work on the worst-case scenario for our analysis.

The remaining set includes 139956 trips and 22750 OD pairs.

#### 3.2.2. Calculation of the Trip Index

For a given trip *p* from origin *i* to destination *j*, the calculation of the trip index $\alpha_{p}$ defined in Equation 4 involves the lengths of four trajectories: the actual trajectory of the trip, $d_{p}$; the linear trajectory, $d_{L}$; the orthodox trajectory, $d_{O}$; and the heterodox trajectory, $d_{H}$.

The actual trajectory of the trip is retrieved from the track data in the BiciMAD database. The linear trajectory is built as the segment connecting the origin *i* to the destination *j*. On the other hand, the orthodox trajectory is retrieved from Google Maps, selecting *bicycle* as the transport mode. This way, the orthodox trajectory is defined by a polygonal curve that includes *n* intermediate points.

To calculate the Fréchet distance from the orthodox (*O*) to the linear (*L*) trajectories, we construct a linear interpolation of *n* segments that link origin *i* to destination *j*. Next, we apply the calculation of the discrete Fréchet distance $\delta_{DF}\left(O,L\right)$ specified in Equation 2 to these two polygonal curves.

Finally, the length of the heterodox trajectory is calculated from the linear and the orthodox trajectories as defined in Equation 3.

### 3.3. Results of the Classification of BSS Trips

We represented the classification of BSS trips as a data-driven problem given the lack of a previously well-defined set of trips falling on either category: transport or leisure. Consequently, we classify BSS trips according to the results obtained from applying the trip index to the specific set of empirical data, and analyzing their inherent features.

**Figure 1:**
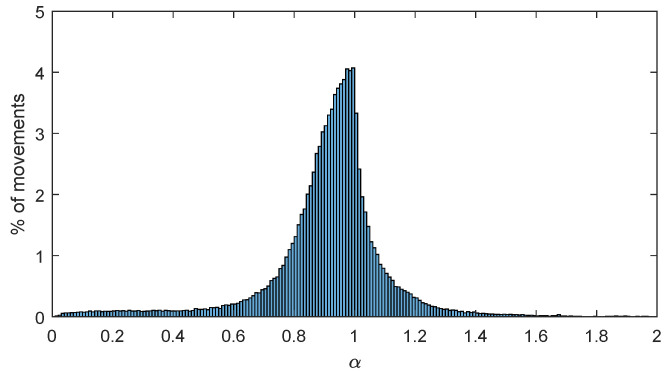
Histogram of trip indexes of trips in the dataset.

Figure 1 shows the histogram of the trip indexes corresponding to the 139956 trips registered in February 2019, in BiciMAD, resulting from the preprocessing phase. We observe two clear behaviors of the trip index, which suggests a concatenation of uniform and Gaussian distributions.

Please note that there are trip indexes greater than 1, which correspond to trips achieving deviations under the Fréchet distance of the orthodox trajectory to the linear path. This fact is perfectly consistent with the proposed methodology. On the other hand, it opens up a new set of questions regarding the actual statistical distribution of the shortest available routes between two points in a BSS, to be specifically addressed in a particular research line that will be discussed in Section 5.

**Figure 2:**
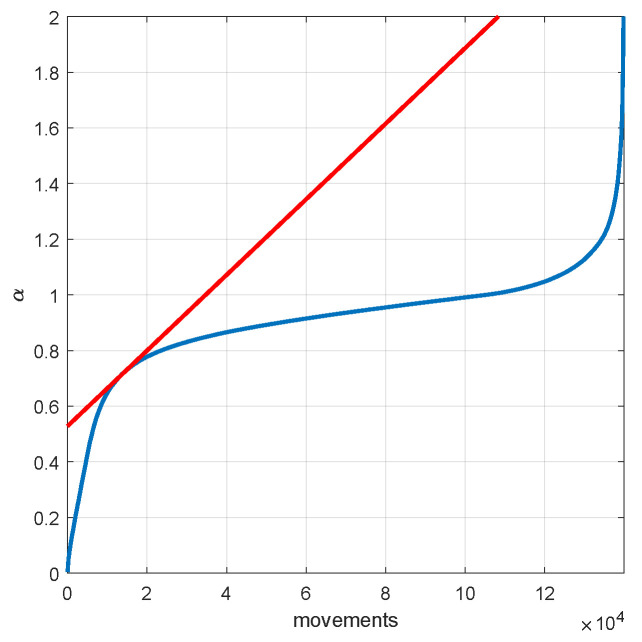
In blue: trip indexes sorted in ascending order; in red: the tangent line obtained by the elbow method.

To determine the optimal threshold that separates each of these two behaviors, we use the *elbow method*, commonly employed in data-driven clustering models. In this respect, Figure 2 shows the trip indexes sorted in ascending order.

The optimal value for the threshold of the trip index is $\alpha^{*}=0.7$. We then use this value to classify trips as transport or leisure.

### 3.4. Validation of the Results

The characterization of mobility often lacks a solid ground-truth [30]. In these cases, we must rely on the analysis of a set of features shown by each resulting group and test whether they actually represent distinct behaviors.

Consequently, in this work, we perform two different analyses to validate the classification methodology based on the trip index: statistical and operational.

#### 3.4.1. Statistical Analysis

**Figure 3:**
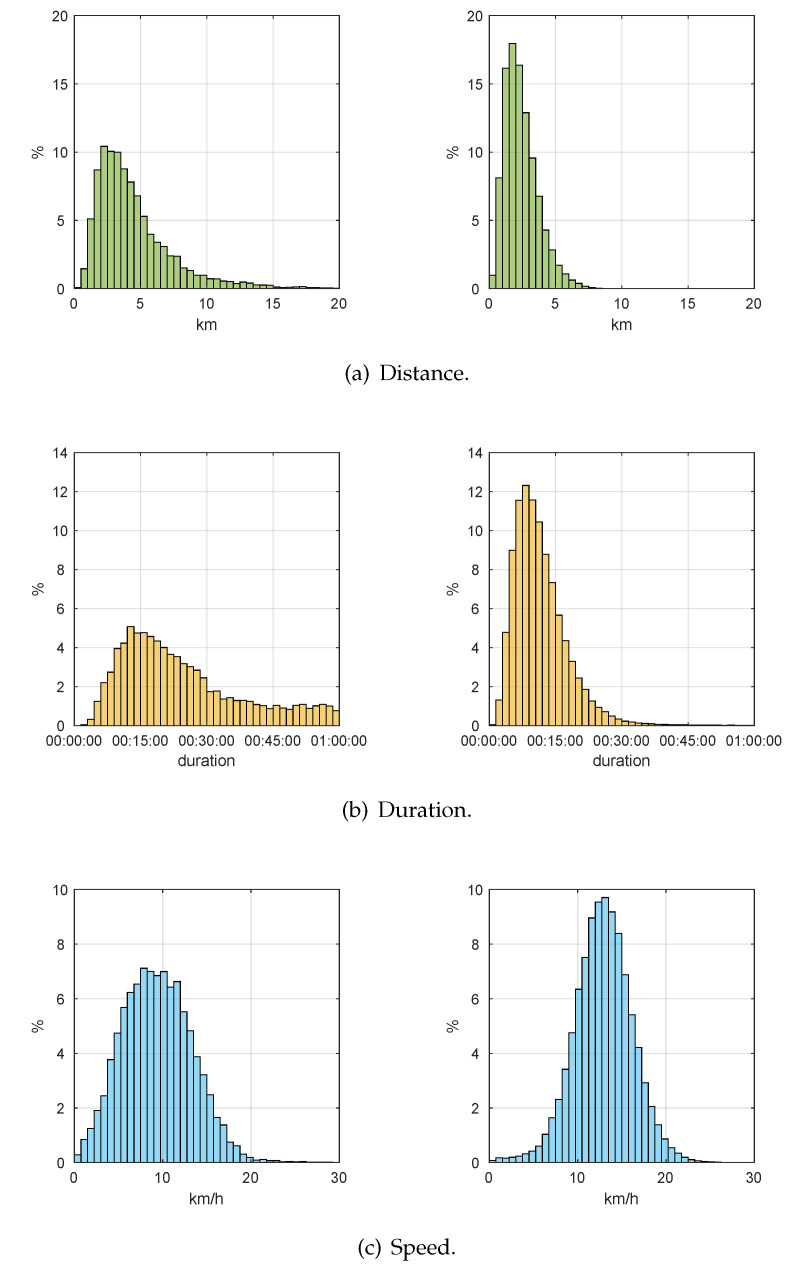
Statistical characterization of trips: *leisure* ($\alpha_{p}<\alpha^{*}$) on the left, and *transport* ($\alpha_{p}\geq \alpha^{*}$) on the right.

First, we complete the statistical analysis of three defining features of the trip: distance, duration, and speed. Figure 3 shows the results we obtained.

We can observe that the shapes of the statistical distributions corresponding to *leisure* (left) and *transport* (right) trips are fundamentally different. Let us study in some depth each of these features and compare them to similar scientific results obtained from BSSs.

Traveled distances in leisure and transport trips are clearly different as they show particular ranges, average values and standard deviations as we can observe in Table 1. Leisure trips are characterized by significantly higher maximum distances, which reflect the user is *wandering*. This result agrees with the conclusions in [31] where authors state that *daily members* of a BSS (leisure) perform longer trips as opposed to *annual members* (transport). In addition, the average distances we obtained fit those resulting from the *non-commute* and *commute* trips in [32].

The specific statistical values of the distribution of the duration of the trip are included in Table 2. In this case, the ranges are similar, but we can observe significant differences among the average and standard deviations. Transport trips are typically shorter in time, which coincides with the results in [32], where the authors conclude that duration of BSS trips is highly correlated with distance. In addition, the authors in [33] affirm that commuters spend 1 hour a day total in their outward and return trips; in our study, Figure 3b shows that the tail of the distributions of duration of trips is negligible beyond 30 min.

Finally, Table 3 shows the numerical results for the distribution of speeds. We can observe a significantly higher average speed in transport trips. This is particularly relevant as bicycles in BiciMAD are electric, which avoids the speed being biased by the physical condition of the user. Higher speeds were also observed in commuters in [32].

#### 3.4.2. Operational Analysis

In addition to the statistical analysis, we perform a second validation on the results of the classification of trips. In this case, we focus on the docking stations that generated or absorbed most of the trips of each kind.

**Figure 4:**
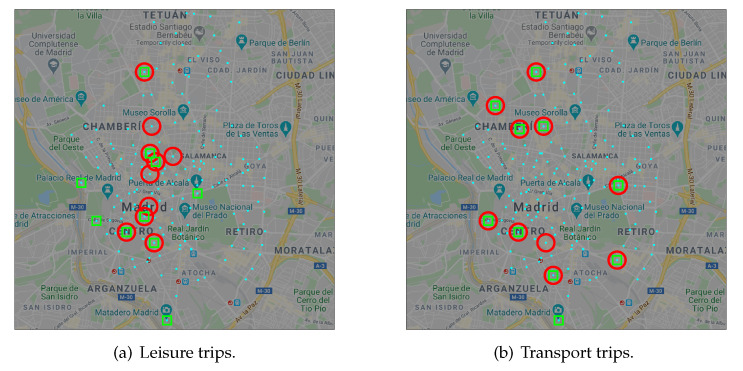
Main origins (red circles) and destinations (green squares) of trips.

Figure 4 shows the top 10 docking stations that acted as origin (red circles) or destination (green squares) of leisure (Figure 4a) and transport (Figure 4b) trips. Leisure trips have destinations located in Madrid’s historic center and some important tourist sites like Puerta de Alcalá (right most green square); Madrid Río (left most green square), a 7-kilometers linear park along the Manzanares river; Matadero de Madrid (bottom most green square) that offers avant-garde cultural exhibitions and performance; or Mercado de Maravillas (top most green square), one of the biggest markets in Europe. On the other side, leisure trips have origins at docking stations mainly located on the historic center and close surroundings. This matches a typical behavior of tourists, who often choose accommodations close to the tourist sites. However, 40% of the top origins and destinations of leisure trips do not share a common docking station; this means that they can be seen as one-way trips. This behavior is opposite to what we observe in transport trips (Figure 4b), where we find 9 docking stations that are simultaneously origin and destination. This matches the usual commuter behavior: from residence to work and back.

We recognized this same behavior on the docking stations activity profile [34], i.e., the net flow of bicycles. Selecting 2 as the number of groups to be generated by the proposed clustering algorithm resulted in two distinct classes of docking stations corresponding to commuter and non-commuter behaviors. In the present work, we reach analogous profiles facing the problem from a different perspective: trajectories instead of docking stations.

This operational analysis confirms that the classification effectively separates trips of either category.

## 4. Underlying BSS Public Transport Network

A public transport system relies on a multiplex network in which different transport modes complement one another. An optimally designed public transport network should provide full coverage, which ideally would connect any pair of points in the space [35]. BSSs provide a public transport network with virtually full coverage, as users can travel the city in a single transport mode. In addition, it may contribute to reduce the travel time avoiding traffic congestions and crowded stops [36]. Furthermore, BSSs add a second functionality to the network: leisure, which is not found in other transport modes.

However, the flexibility in choosing routes throughout a BSS complicates the extraction of its underlying network. The classification methodology we propose allows us to identify the set of trips that were performed using the BSS as a transport mode or just for leisure. Consequently, we can derive the underlying BSS public transport network from the trajectories of these trips and study their fundamental purpose.

**Figure 5:**
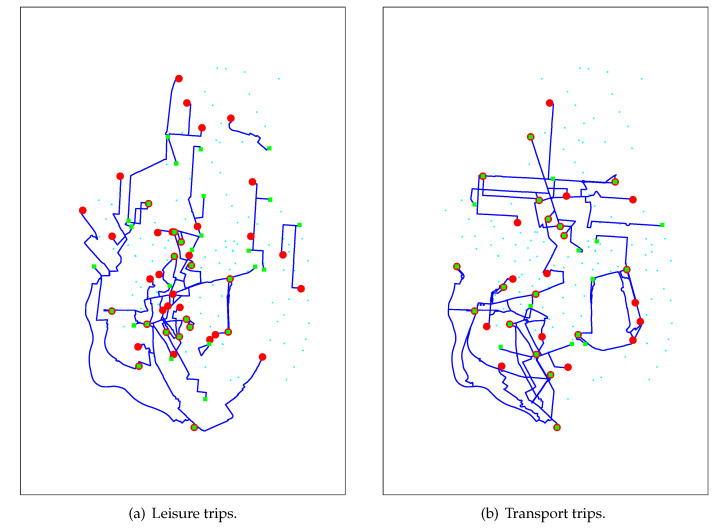
Main leisure and transport trajectories.

Figure 5 shows the trajectories of the 50 OD pairs that account for the greater number of leisure and transport trips. Leisure trips (Figure 5a) required 61 docking stations in order to reach this top 50 OD pairs, while transport trips (Figure 5b) needed only 40. Merging these two particular networks, we can infer the overall BSS transport network.

As we can observe, leisure and transport networks show significant differences in structure: dispersion, compactness, loose links, etc. To highlight these differences, let us consider the complete set of leisure and transport trips, which form the underlying network of each category. These two networks can be analyzed as graphs, calculating their density. The corresponding results are shown in Table 4.

Every docking station in the BSS (169) act as origin or destination of trajectories in both networks. Consequently, their density must be lower than that of the total network. As we can observe, the variation in density of the leisure network to the total network is significantly higher (−66.25%) than the one corresponding to the transport network (−3.75%). This fact demonstrates the distinct underlying structure of both networks.

**Figure 6:**
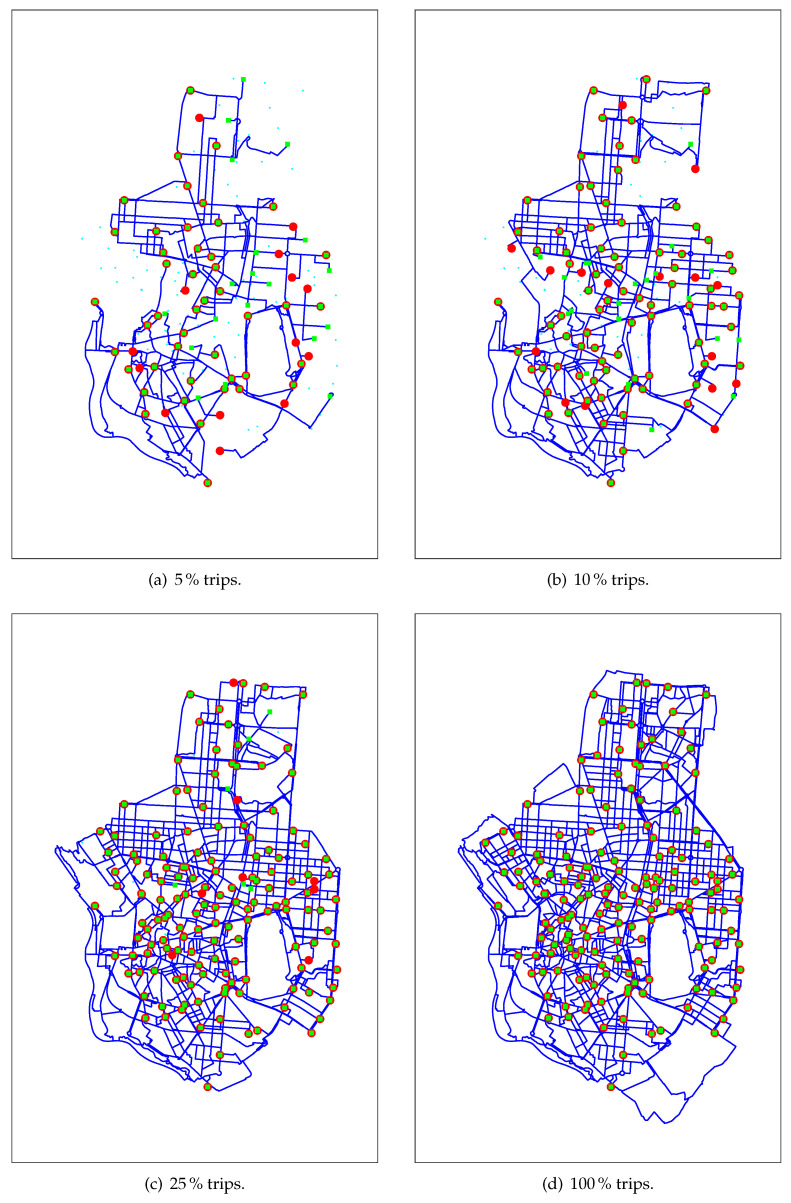
Formation of BiciMAD public transport network.

Let us now focus on the underlying network induced by transport trips. Figure 6 shows the sequential formation of this network considering an increasing percentage of trips. Observing Figure 6d we can clearly state that BiciMAD’s underlying public transport network provides almost full coverage (most blank spaces correspond to parks). In addition, this analysis provides meaningful information about the importance of every route in the network. This information could build a solid basis for support decision tools that would help municipalities in the design of new infrastructures or changes in the urban regulation to promote the use of BSSs.

Finally, we can conclude that 90.60% of BiciMAD’s trips were performed as a public transport mode, which highlights the significant contribution of BSSs to public transport in a city.

## 5. Conclusions and Future Research

This work proposes a methodology to classify trips depending on their intrinsic purpose: transport or leisure. This classification is based on the trip index, which measures the actual travel distance versus the available shortest path. In a BSS, this is not a straightforward measure given that cyclists may use shortcuts that are not contemplated in the underlying network (streets and bicycle lanes) and the regulatory restrictions. In addition, these shortcuts have a time-to-live. Consequently, we constructed a mathematical framework to estimate the heterodox trajectory and take it as the reference for the trip index.

We validated the classification methodology empirically, using data from BiciMAD and analyzing the results from both statistical and operational perspectives.

The analysis of trajectories in BSSs opens a set of future research lines. First, in our study we have found trip indexes greater than 1. This implies that some users achieved trajectories that showed a deviation from the linear path below the Fréchet distance of the orthodox trajectory. This means that under certain circumstances, we can find shortcuts to reduce the traveled length in a BSS. The detailed study of the spatio-temporal existence of these short cuts will be addressed in a specific research.

This analysis will use the Fréchet distance to show the deviation of each actual trip to the linear path between origin and destination. The resulting set of deviations will form a probability distribution, which will eventually model the overall *directivity* of trajectories in a particular BSS. This new concept embeds information regarding both the user and the underlying topology. Thus, using the proposed methodology to restrict the analysis to transport trips will lead us to a global characterization of BSS networks. The resulting methodology will then be applied to datasets of BSS trajectories in different cities to compare their relative performance as a public transport mode.

In addition, trajectories will also be used to predict future occupancy levels in docking stations. This research will be based on a metaheuristic approach based on swarm intelligence, which will characterize how groups of bicycle users flow from one docking station to another.

On the other hand, identifying users’ behavior plays a key role in understanding their needs to provide them with optimized services. Companies invest huge amounts of money a year for this type of findings. Public institutions such as municipalities find this knowledge even more important, given that they must select where to invest public money so that they provide the best services to their citizens. Importantly from our perspective, it provides foundation information for planning and design, which will lead to optimization of the deployment of this type of transport mode. Our work provides mathematical and empirical evidence on the type of users a BSS has. This allows municipalities to configure tariffs to promote this type of transport, invest in new bicycle lanes that follow the actual routes users are traveling, etc.

In this respect we have demonstrated that BSSs are a form of public transport not only for the last mile, but in its own right. Consequently, municipalities may start considering them in more depth as a solution for urban transport. Our methodology provides a framework to generate meaningful information to be employed as a decision support tool to the process of reengineering the bicycle infrastructure and the corresponding regulation. This information builds a solid ground of knowledge for BSS managers and municipalities.

## Figures and Tables

**Table 1:** Distance Statistics (km).

	Min.	Max.	Mean	Std. Dev.
**Leisure** ($\alpha_{p}<\alpha^{*}$)	0.3	47.9	4.5	3.0
**Transport** $\left(\alpha_{p}\geq \alpha^{*}\right)$	0.1	10.0	2.4	1.2

**Table 2:** Travel Time Statistics.

	Min.	Max.	Mean	Std. Dev.
**Leisure** $\left(\alpha_{p}<\alpha^{*}\right)$	00:02:22	05:57:57	00:37:20	00:38:22
**Transport** $\left(\alpha_{p}\geq \alpha^{*}\right)$	00:00:58	05:59:26	00:11:56	00:09:52

**Table 3:** Speed Statistics (km/h).

	Min.	Max.	Mean	Std. Dev.
**Leisure** $\left(\alpha_{p}<\alpha^{*}\right)$	0.3	28.7	9.4	3.9
**Transport** $\left(\alpha_{p}\geq \alpha^{*}\right)$	0.2	29.4	12.9	3.3

**Table 4:** Comparison of Networks.

	Total	Leisure	Transport
**trips**	139956	13156	126800
**order**	169	169	169
**size**	22750	7617	21947
**DENSITY**	0.80	0.27	0.77
